# KLF2 inhibition expands tumor-resident T cells and enhances tumor immunity

**DOI:** 10.21203/rs.3.rs-5966555/v1

**Published:** 2025-03-13

**Authors:** Eli Gilboa, Vineet Gupta, Darija Muharemagic, Sunwoo Ham, Erietta Stelekati, Emily Clark

**Affiliations:** University of Miami; University of Miami; University of Ottawa; University of Miami; University of Miami, Florida, USA

## Abstract

Tissue resident memory CD8+ T cells (Trm) constitute a distinct population of non-circulating memory T cells^1–5^ vastly exceeding the number of circulating T cells^5^, and play a pivotal role in protective immunity against pathogens^6–8^. How to promote the generation of vaccine specific Trm remains an important challenge. Whether Trm contribute also to immune control of tumors or just correlate with an unrelated process linked to clinical outcome has not been unequivocally established^9,10^, and phenotypic markers such as co-expression of CD69 and CD103 or CD49a integrins commonly used to monitor tumor infiltrating Trm do not unambiguously define this subset. Here we tested the hypothesis that transient downregulation of KLF2, the most conserved feature of Trm ontogeny^4,11,12^, will promote the differentiation of vaccine activated CD8+ T cells into Trm and enhance antitumor immunity. We show that 4-1BB antibody targeted delivery of a KLF2 siRNA to tumor bearing mice led to the downregulation of KLF2 in vaccine activated CD8+ T cells and the accumulation of phenotypically defined intratumoral CD69+CD103+ and CD69+CD49a+ CD8+ T cells which correlated with enhanced control of tumor growth. This study could serve as the foundation of a broadly applicable and clinically useful way to promote the generation of vaccine specific Trm and provides direct evidence that intratumoral CD8+CD69+CD103+ and CD8+CD69+CD49a+ cells are indeed Trm and that Trm contribute to tumor immunity.

## INTRODUCTION

Murine and human studies have highlighted the importance of persistence of the vaccine-induced or adoptively transferred T cell mediated immunological memory in mediating protective immunity in the setting of infectious diseases and cancer^13–15^. Memory T cells were thought to patrol peripheral tissues by constant recirculation between tissues and secondary lymph organs. Recent studies have shown that most memory T cells become sequestered in peripheral tissues constituting a distinct population of non-circulating memory T cells called tissue resident memory T cells (Trm)^1–4^, exceeding the number of circulating T cells^5^.

Several lines of evidence suggest that Trm have a pivotal role in protective immunity against pathogens and tumors^1–4,16,17^. Adoptive transfer^6^, parabiosis^7^, and tissue entrapment^8^ experiments have provided direct evidence that in preclinical murine models of HSV, influenza, and vaccinia virus infection Trm provide superior protection from reinfection compared to circulating memory T cells. In cancer patients, tumor infiltrating phenotypically defined CD103+CD8+ Trm cells were a better predictor of survival than CD8+ T cells, and the density of CD8+ T cells with a Trm transcriptional signature correlated with survival and immunotherapy response(reviewed in^10^). In mice, genetic modifications that promote Trm differentiation improved antitumor efficacy of adoptive T cell therapies^18–20^, and vaccination protocols that promote increased Trm generation correlated with enhanced antitumor efficacy^21–27^. Development of broadly useful therapeutic strategies to promote the differentiation of vaccine-induced T cells into Trm could therefore significantly improve immune based therapy.

Correlative studies of tumor infiltrating Trm and survival^10^, forced generation of Trm^18–20^, and vaccine efficacy^21–27^ are short of definitive proof for the role of Trm in tumor immunity^9,10^. The hallmark of Trm is tissue residency. The existence of pathogen-specific noncirculating Trm and their pivotal role in protective immunity has been firmly established by parabiosis experiments in mice^7^. Demonstrating the presence of tumor infiltrating Trm by measuring their migratory properties, especially in short lived transplantable murine tumors, is experimentally challenging^9,10^. Tumor resident Trm in mice and humans are, therefore, generally monitored using phenotypic markers like CD8+CD69+CD103+ or CD8+CD69+CD49a+ T cells. Since not all Trm express these markers and their expression is not restricted to Trm the question remains whether such phenotypically defined T cells are bona fide Trm. Moreover, to what extent such phenotypically defined Trm are responsible for tumor control or just correlate with the immunological status of the tumor to promote a robust antitumor response has not been unequivocally demonstrated^9,10^.

Downregulation of the transcription factor KLF2 is the most conserved molecular feature of Trm, establishing their tissue or tumor residence^4,12^. A main target of KLF2 is S1PR1 the receptor for the endothelial derived S1P (sphingosine-1-phosphate) which promotes egress of T cells from peripheral tissues and lymph nodes^11^. In this study we tested the hypothesis that transient downregulation of KLF2 in vaccine-activated CD8+ T cells will promote their differentiation into Trm and enhance tumor control, thereby providing direct evidence for the role of Trm in tumor immunity and introducing a broadly useful approach to promote vaccine induced Trm, not limited to cancer.

### KLF2 downregulation in activated CD8+ T cells.

**Figure 1:**
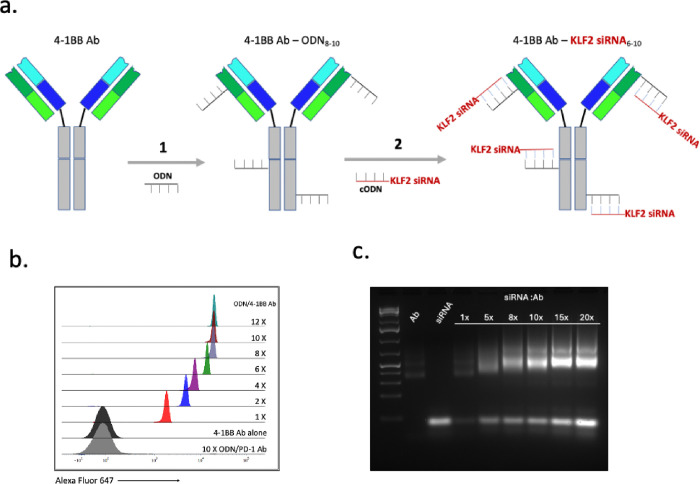
Conjugation of KLF2 siRNA to a 4-1BB antibody. **a.** Step 1, a 19 nt 2’-O-methyl (2’OMe)-modified ribooligonucleotide (ODN) is conjugated to 8–10 alpha amino groups of about 40 accessible lysines using a stable bis-aryl hydrazone bond (mAb-ODN). Step 2, a complementary ODN (cODN) is attached to the 3’ end of the sense strand of the KLF2 siRNA and hybridized to the ODN-modified antibody in PBS at 1:1 ratio. For additional details see Methods and reference^28^. **b.** Valency of the antibody-ODN. The average number of ODNs conjugated to the antibody was determined by hybridization of a fluorophore-labeled complementary ODN. **c** Monitoring hybridization reaction by agarose gel analysis. ODN conjugated Clec9a antibody was hybridiized with TAP siRNA (siRNA) at increasing molar ratios of siRNA to ODN, run on a 2% agarose gel and visualized by UV. siRNA bindiing was saturated between 10–12 siRNA molecules per antibody. The antibody-siRNA conjugate is aliquoted and stored at 4 °C for at least 30 days.

To downregulate KLF2 in recently activated CD8+ T cells we used a modular antibody targeting platform whereby multiple copies of a KLF-2 specific siRNA were conjugated to a 4-1BB antibody as described in reference^28^, and shown in Fig. 1. Briefly, 8–10 copies of a 19 nt long oligonucleotide (ODN) were chemically conjugated to a 4-1BB antibody, and a second step KLF-2 or control siRNA was conjugated to the ODN-modified antibody by hybridization via an attached complementary sequence (cODN), resulting in a multivalent configuration whereby the 4-1BB antibody delivers multiple siRNA molecules to 4-1BB expressing vaccine activated CD8+ T cells. A fluorophore-labeled complementary ODN was used to determine the average number of ODNs conjugated per antibody (Fig. 1b) and the hybridization of the siRNA to the ODN-modified 4-1BB antibody was monitored by agarose gel analysis (Fig. 1c)

**Figure 2:**
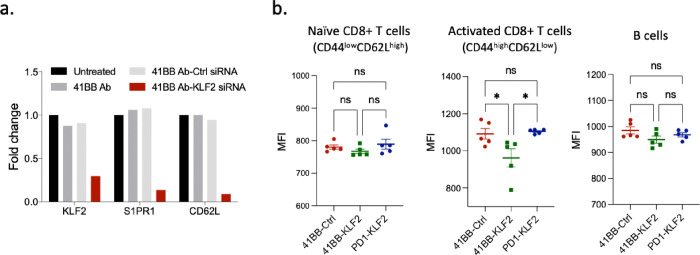
4-1BB antibody targeted delivery of a KLF2 siRNA leads to the downregulation of KLF2 in activated CD8+ T cells. **(a)** CD8+ T cells isolated from spleen were polyclonally activated with CD23/CD28 antibodies and incubated with 4-1BB antibodies, 4-1BB antibodies conjugated to a KLF2 or control siRNA. Downregulation of KLF-2, S1PR,1 and CD62L was determined by qRT-PCR. **(b)**. Mice were vaccinated against the TAP downregulation induced antigens using a TAP siRNA conjugated to a CpG ODN which targets the siRNA to and downregulates TAP in resident dendritic cells as described in reference^32^. 24 hours later the vaccinated mice were treated with 4-1BB-KLF2 siRNA, 4-1BB-Ctrl siRNA or a PD-1 antibody conjugated to KLF-siRNA. Naive CD8+ T cells, activated CD8+ T cell and B cells were isolated from the spleen (Extended data Fig. 1) and the expression of KLF2 was determined in each mouse by flow cytometry. Statistical analysis of activated CD8+ T cells (middle panel): 4-1BB-KLF2 vs. 4-1BB-Ctrl or PD-1-KLF2, p=0.0404 and 0.0250, respectively. ns-not significant.

Activated CD8+ T cells transiently upregulate 4-1BB on their cell surface^29,30^. Fig. 2a shows that polyclonally activated CD8+ T cells incubated in vitro with 4-1BB antibody conjugated KLF2 siRNA (4-1BB-KLF2 siRNA), but not control, siRNA led to KLF2 as well as S1PR1 and CD62L downregulation. Downregulation of the nontargeted S 1PR1 and CD6L RNAs is suggestive of the physiological relevance of the siRNA mediated reduction in KLF2 expression since S1PR1 and CD62L are downstream targets of KLF2 whose expression is reduced in Trm compared to circulating antigen activated CD8+ T cells^31^.

To determine whether systemic administration of 4-1BB-KLF2 siRNA to mice leads to KLF2 downregulation in activated CD8+ T cells in vivo, mice were first vaccinated against TAP downregulation induced antigens by treatment with a CpG oligonucleotide targeted TAP siRNA as previously described^32^, and then treated systemically with 4-1BB-KLF2 or control siRNA. Naïve (CD44l^ow^CD62L^high^), activated (CD44^high^CD62^low^) CD8+ T cells and B cells were isolated from the spleen and analyzed for KLF-2 expression by flow cytometry. In this and subsequent experiments mice were treated with 4-1BB-KLF2 siRNA 24 hours post vaccination in order to prevent or reduce the trapping of vaccine activated CD8+ T cells in lymph nodes^33^. While conditions were not optimized, in preliminary experiments delaying 4-1BB-KLF2 siRNA administration for 48 hours did not appreciably affect Trm accumulation in the spleen or tumor tissue (data not shown). Fig. 2b shows that activated CD8+ T cells, but neither naive CD8+ T cells nor B cells, exhibited a significant reduction in KLF2 expression, consistent with the selective 4-1BB targeting of the KLF2 siRNA to 4-1BB expressing activated CD8+ T cells. Cumulatively, these experiments show that treatment with 4-1BB antibody conjugated KLF2 siRNA downregulates KLF2 expression in activated CD8+ T cells both in vitro and in vivo, and that treatment of vaccinated mice with systemically administered 4-1BB-KLF2 siRNA leads to the preferential downregulation of KLF-2 in vaccine activated CD8+ T cells, presumably enriched for T cells with specificities encoded by the vaccine formulation.

### Intratumoral accumulation of Trm.

**Figure 3:**
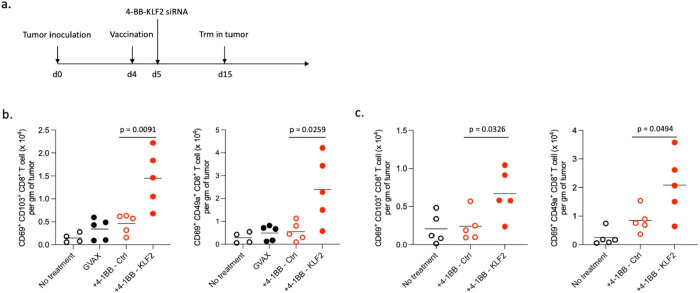
Treatment with 4-1BB-KLF2 siRNA leads to increased accumulation of intratumoral CD8+ Trm (**a).** Experimental design. Mice were implanted subcutaneously with tumor cells, vaccinated at day 4 post tumor implantation, and 24 hours later treated with 4-1BB-KFL2 or 4-1BB Ctrl siRNAs administered by intraperitoneal injection. At day 15 tumors were isolated and the number of CD69+CD103+ or CD69+CD49a+ CD8+ Trm per gm of tumor was determined by flow cytometry as shown in Extended data Fig. 2. **(b)** Mice were implanted with B16.F10.9 tumor cells and vaccinated with GM-CSF expressing irradiated tumor cells (GVAX). **(c)**. Mice were implanted with TAP deficient RMA-S tumor cells and vaccinated with CpG-TAP siRNA as described in Fig. 2b.

To determine whether 4-1BB targeted KLF2 downregulation leads to intratumoral accumulation of Trm, tumor bearing mice were first vaccinated and then treated with 4-1BB-KLF2 siRNA. 15 days following tumor implantation the density of intratumoral CD8+ CD69+CD103+ and CD8+CD69+CD49a+ Trm was determined by flow cytometry (Fig. 3a). Fig. 3b shows that administration of 4-1BB-KLF2, but not 4-1BB-Ctrl, siRNA to B16/F10.9 tumor bearing mice vaccinated with GM-CSF expressing irradiated tumor cells (GVAX) led to increased accumulation of Trm in the tumor tissue. Similarly, Fig. 3c shows that treatment with 4-1BB-KLF2 siRNA led to increased accumulation of intratumoral Trm in mice bearing TAP deficient RMA-S tumors vaccinated against the class I restricted neoepitopes induced by TAP downregulation as described in reference^32^. Since KLF2 downregulation is a defining feature of Trm generation and maintenance^4,11,12^, these observations provide direct evidence that the intratumoral phenotypically defined CD8+CD69+CD103+ or CD8+CD69+CD49a+ cells are indeed Trm.

### Trm enhance tumor immunity.

**Figure 4:**
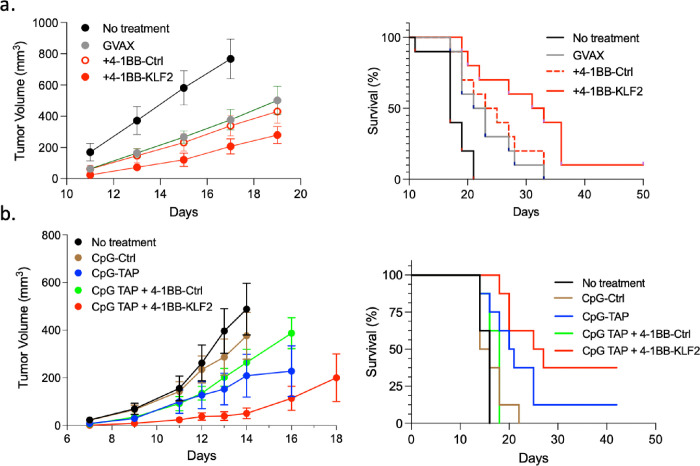
Treatment with 4-1BB-KLF2 siRNA potentiates vaccine-induced antitumor immunity. **(a)** Mice bearing a subcutaneously implanted B16.F10.9 tumor were vaccinated with GM-CSF expressing irradiated tumor cells (GVAX) and treated with either 4-1BB-KLF2 (+4-1BB-KLF2) or with 4-1BB-Ctrl (+4-1BB-Ctrl) siRNAs. Tumor progression (left panels) and survival (right panels). Statistical analysis. Survival (right panel), GVAX + 4-1BB-KLF2 versus no treatment, p<0.001, GVAX + 4-BB-KLF2 versus GVAX + 4-1BB-Ctrl p=0.0442. Tumor progression (left panel), GVAX + 4-1BB-KLF2 versus no treatment d11 p=0.097, d13 p=0.0036, d15 p=0.0008, d17 p=0.0005; GVAX versus GVAX + 4-1BB-KLF2, d11 p=0.0111, d13, p=0.0218, d15 p=0.0224, d17 p=0.0508, d19 p=0.047. **(b)** Same as in panel a using TAP deficient RMA-S tumor bearing mice vaccinated with CpG-TAP siRNA^32^. Statistical analysis. Survival (right panel) +4-1BB-KLF2 vs. +4-1BB-Ctrl), p=0.0008; CpG-TAP versus +4-1BB-KLF2, p=0.1688. Tumor progression (left panel). +4-1BB-KLF2 versus no treatment, d7 p=0.0499, d9 p=0.0239, d11 p=0.0266, d13 p=0.0023, d14, p=0.0062; +4-1BB-KLF2 versus CpG-TAP, d7 p=0.01, d9, p=0.0104, d11, 0.0131, d13 p=0.007, d14 p=0.0062; +4-1BB-KLF2 versus +4-1BB-Ctrl, d12 p=0.092, d13 p=0.0016, d14 p=0.0031, d16, p=0.005.

We next tested whether 4-1BB targeted KLF2 siRNA treatment can enhance vaccine induced antitumor immunity using the B16/F10.9 and RMA-S tumor models described above. The dose of the agonistic targeting 4-1BB antibody was calibrated to minimize its contribution to tumor inhibition. As shown in Fig. 4, in both models, systemic administration of 4-1BB antibody targeted KLF2 (+4-1BB-KLF2), but not 4-1BB targeted control (+4-1BB-Ctrl), siRNA enhanced vaccine-induce antitumor immunity, measured as tumor progression and survival. Note that while the difference in survival of the RMA-S tumor bearing mice vaccinated with CpG-TAP siRNA and treated with 4-1BB-TAP siRNA (+4-1BB-TAP) was statistically significant compared to treatment with 4-1BB-Ctrl siRNA (+4-1BB-Ctrl) (p=0.008), it failed to reach statistical significance when compared to mice that were only vaccinated with CpG-TAP siRNA (CpG-TAP)(p=0.1688). A likely explanation is that in the RMA-S model the 4-1BB antibody conjugated to control or TAP siRNA reduced the vaccine (CpG-TAP) induced antitumor response (Fig. 4b, compare CpG-TAP and +4-1BB-Ctrl groups), conceivably due to a suppressive effect of the 4-1BB antibody.

To determine the contribution of adaptive CD4+ and CD8+ T cell responses to KLF2 siRNA mediated antitumor immunity, GVAX vaccinated mice treated with 4-1BB-Ctrl or 4-1BB-KLF2 siRNA were depleted of CD4 or CD8 cells and monitored for tumor progression (Extended Data Fig. 3a & 3c) and survival (Extended Data Fig.3b & 3d). Treatment of the vaccinated mice with 4-1B-KLF2 siRNA significantly inhibited tumor growth compared to untreated mice or mice treated with 4-1BB-Ctrl siRNA. Depletion of CD8+ T cells abrogated the antitumor effect of 4-1BB-KLF2 siRNA but had no measurable impact on mice treated with 4-1BB-Ctrl siRNA (Extended Data Fig. 3a & 3b), showing that CD8+ T cells were pivotal in the KLF2 siRNA mediated antitumor response and suggesting that the small antitumor effect of the targeting agonistic 4-1BB antibody was mediated primarily by non-T cells. In contrast, depletion of CD4 cells enhanced the antitumor response in mice treated with either 4-1BB-KFL2 siRNA or 4-1BB-Ctrl siRNA, showing that CD4+ T cells exerted an immune suppressive effect (Extended Fig. 3d), also explaining in part the apparent lack of CD8+ T cell contribution to the small antitumor response of 4-1BB-Ctrl siRNA. Of note, the suppressive effect of CD4+ T cells was significant when survival (Extended Data Fig. 3d), but not when tumor progression (Extended Data Fig. 3c), was measured, conceivably because monitoring of tumor progression was halted when 2 or more mice reached maximum tumor size in the untreated group. The nature of the suppressive CD4+ cells, whether Foxp3+ Treg or Tr-1 cells has not been determined.

Taken together, the experiments described in Figs. 2–4 and Extended Fig. 3 show a direct correlation between downregulation of KLF-2 in vaccine-activated CD8+ T cells in vivo (Fig. 2b), increased number of intratumoral Trm cells (Fig. 3), and enhanced antitumor immunity (Fig. 4), supporting a pivotal role for Trm in protective antitumor immunity and providing the basis of a broadly applicable way to promote the generation of vaccine activated Trm.

## DISCUSSION

In this study we describe a general way how to promote the differentiation of vaccine activated, and hence antigen specific, CD8+ T cells into Trm that leads to enhanced protective antitumor immunity in mice. We show that 4-1BB antibody targeted delivery of a KLF2 siRNA to vaccine activated CD8+ T cells in tumor bearing mice leads to increased intratumoral content of Trm cells (Fig. 3) and enhances vaccine mediated inhibition of tumor growth (Fig. 4). The approach to promote Trm differentiation is readily applicable also to infectious diseases and adoptive T cell therapy.

Given the mounting evidence for the role of Trm in protective immunity, development of broadly useful therapeutic strategies to promote the generation of vaccine-induced Trm could significantly improve immune based therapy. Shin and Iwasaki described an approach whereby vaccine induced CD8+ T cells are recruited and sequestered at the site of infection by topical administration of chemokines^8^. This approach is dependent on accessible tissues or tumor lesions for chemokine administration posing a significant impediment for reaching and impacting disseminated metastatic lesions. The approach described in this study of targeted downregulation of KLF2 in activated CD8+ T cells using a systemically administered drug formulation, the 4-1BB antibody conjugated KLF2 siRNA, will reach activated CD8+ T cells throughout the body and hence improve immune control of tumor lesions wherever they may be.

Therapeutic targeting of the Trm compartment is predicated on the premise that Trm contribute to protective immunity. However, to what extent Trm are responsible for tumor control or just correlate with the immunological potential of the tumor microenvironment to promote a robust antitumor response has not been unequivocally demonstrated^9,10^. This is further complicated by the fact that tumor infiltrating CD8+ Trm are generally monitored using phenotypic markers, most commonly cell surface co-expression of CD69 and the CD103 or CD49a integrins, as used in this study. Since not all Trm express these markers and their expression is not restricted to Trm the question remains whether such phenotypically defined T cells are bona fide Trm. Whereas the hallmark of Trm is tissue or tumor residency, measuring the migratory properties of tumor infiltrating CD8+ T cells using parabiosis experiments, especially in short lived transplantable murine tumors, is challenging. Experiments using FTY 720 have shown that recently activated lymph node derived T cell do not make or make a limited contribution to antitumor immunity^21,34^, suggesting that noncirculating tumor resident T cells play a key role in controlling tumor growth. These experiments, however, did not determine whether the protective T cells consisted of noncirculating tumor resident cells or circulating T cells derived from nonlymphoid tissue, nor have they identified the nature of the T cells, whether they corresponded to the phenotypically defined CD69+CD103+ and/or CD69+CD49a+ canonical Trm. Here we show that experimental downregulation of KLF2 in vaccine activated CD8+ T cells (Fig. 2) led to the accumulation of intratumoral phenotypically defined CD69+CD103+ and CD69+CD49a+ Trm (Fig. 3), which correlated with enhanced inhibition of tumor growth (Fig. 4). Since KLF2 downregulation in antigen experienced CD8+ T cells is also a defining feature of Trm generation and maintenance^4,11,12^, these observations provide the most direct evidence to date that the intratumoral phenotypically defined CD8+CD69+CD103+ or CD8+CD69+CD49a+ cells are indeed Trm, and consequently that Trm contribute to inhibition of tumor growth in mice.

The experiments described in Figs. 3 and 4 show that systemic administration of 4-1BB-KLF2 siRNA increases the intratumoral content of tumor-specific Trm and potentiates vaccine-induced antitumor immunity. Notwithstanding, the therapeutic impact though statistically significant was modest, most likely because the experimental conditions of this proof-of-concept study were suboptimal. For example, the optimal timing between vaccination and 4-1BB-KLF2 siRNA administration has not been determined. In our experiments 4-1BB-KLF2 siRNA was administered 24 hours post vaccination to minimize the trapping of the vaccine activated 4-1BB expressing CD8+ T cells in the lymph nodes^33^.

The 4-1BB targeting antibody and the KLF-2 siRNA are prototypes that were used to demonstrate the concept. Future studies to optimize the antibody targeted siRNA platform, such as targeting the siRNAs to PD-1 or CD69 on the vaccine-activated CD8+ T cells, promoting Trm generation by downregulating S1PR1, or optimizing the antibody-siRNA backbone formulation, could also improve the therapeutic potency of this approach.

## METHODS

### Cell Lines:

RMA-S (TAP2-deficient) cells was obtained from T. van Hall (Leiden University Medical Center, Leiden, the Netherlands) and were cultured in Iscove modified Dulbecco’s medium (IMDM) media (Gibco), supplemented with 10% heat-inactivated FBS, penicillin (100 U/mL), streptomycin (100 μg/mL; Gibco Life Technologies),1 mmol/L sodium pyruvate, 0.05 mmol/L β-mercaptoethanol, and 2 mmol/L minimal essential medium (MEM) nonessential amino acids (all from Gibco Life Technologies) in a humidified incubator at 37 °C and 5% CO_2_..

B16-F10.9 cell line was maintained in Dulbecco’s Modified Eagle’s Minimal Medium (DMEM), supplemented with heat-inactivated 10% fetal bovine serum (FBS), penicillin (100 U/mL), streptomycin (100 μg/mL), 25 mM HEPES, 2 mM L-glutamine, and 1 mM sodium pyruvate in a humidified incubator at 37 °C and 5% CO_2_. A cell line derived by transfection of B16/F10.9 cells with a murine GM-CSF cDNA plasmid, F10.9/GM, was used as a source of GM-CSF for generating murine DCs. F10.9/GM cells were grown in RPMI 1640 supplemented with heat-inactivated 10% fetal bovine serum (FBS), penicillin (100 U/mL), streptomycin (100 μg/mL), 1 mM sodium pyruvate, 1 mM nonessential amino acids (NEAA), 0.5mM β-mercaptoethanol and 10 mM HEPES (all from Gibco Life Technologies) in a humidified incubator at 37 °C and 5% CO_2_.

### Generation of the 41BB antibody-oligonucleotide (ODN) conjugates:

Antibody conjugations were performed using a modified protocol from Vector Laboratories for protein-oligonucleotide conjugation. All reagents were sourced from Vector Laboratories unless otherwise specified. The antibody-oligonucleotide calculator provided by Vector Laboratories (https://vectorlabs.com/productattachments/protocol/VL_A-9202-001_Calculator_LBL02104.xlsx?srsltid=AfmBOorAc1NQ4Xe9MkWk9fkPinpvWq7FAv9eesBmxcJNAV-_PInEUS0j) was used throughout the process. Conjugation process was carried out in two steps. First, a 5′ amino-modified oligonucleotide (ODN) was labeled with S-4 formylbenzamide (4-FB). This 4-FB modified ODN was then covalently linked to a S-Hynic labeled antibody using an aniline catalyst to produce a stable antibody-ODN conjugate.

To begin, the 5′ amino-modified ODN was resuspended in 1× Modification Buffer at a concentration of 0.5 OD/μL, diluted 1:200 in water, and the concentration (OD_260_/μL) was determined using a NanoDrop. The volume and concentration were input into the calculator. The ODN was combined with the calculated volume of anhydrous DMF and resuspended S-4-FB and subsequently incubated for 2 hours at room temperature (RT) on a rotator. The 4-FB ODN was then purified and desalted twice into 1× Conjugation Buffer using a 7 K MWCO Zeba Column (Thermo Scientific). After purification, the ODN concentration was measured, aliquoted, and stored at −80 °C.

To conjugate 8 ODN molecules per antibody, the antibody (InVivoPlus anti-mouse 4-1BB (CD137) Clone 3H3; BioXCell) was desalted into 1× Modification Buffer using Amicon Ultra 30 K MWCO centrifugal filters (Millipore), following the manufacturer’s instructions for three buffer exchanges. The antibody was collected, and its protein concentration was measured with a NanoDrop, adjusting it to no more than 2 mg/mL. S-Hynic was resuspended in anhydrous DMF and used to label the antibody at a 50-fold molar excess (1 μg IgG = 6.6 pmol). The antibody was incubated with S-Hynic for 2.5 hours at room temperature on a rotator. After incubation, excess S-Hynic was removed by buffer exchange into 1X Conjugation Buffer using Amicon filters. The S-Hynic-labeled antibody was then incubated with an 8-fold molar excess of 4-FB-modified ODN in 10X Catalyst Buffer and subsequently incubated for 2 hours at room temperature (RT) on a rotator. Following this, the antibody-ODN conjugate was desalted into PBS (without Ca++ or Mg++, Gibco) using Amicon filters. The antibody concentration was determined using the Pierce BCA protein assay (Thermo Scientific) and stored at 4°C for up to one month. For each experiment, complementary ODN (cODN)-modified siRNA was hybridized to the antibody-conjugated ODNs at a 1:8 molar ratio at 41°C for 15 minutes in PBS with divalent cations to yield 8 ODN molecules per antibody.

To confirm ODN conjugation to the antibody and the removal of excess free ODNs, 1 mg of antibody-ODN conjugate was analyzed using 2% agarose gel electrophoresis in TBE buffer, stained with ethidium bromide, and visualized under UV light. Additionally, to assess whether ODN conjugation affected antibody binding, the antibody-ODN conjugates were incubated with 4-1BB-Fc-coated beads, and fluorescence saturation was checked by flow cytometry. To prepare the beads, 10 μL of Protein A DynaBeads (Thermo Scientific) were washed three times with bead buffer (20 mM Hepes, pH 7.4, 10 mM NaCl, 2 mM CaCl₂, 2 mM MgCl₂, 0.02% azide, 0.01% Tween-20). The washed beads were then incubated with 10 μg of murine r4-1BB-Fc chimeric protein (R&D) in 100 μL of bead buffer for 1 hour at room temperature with rotation. The beads were washed again and then incubated with 10 μg of IgG isotype control antibody. After a final wash, the beads were stored in 1 mL of bead buffer at 4°C until use.

To calculate the quantity of ODNs conjugated per antibody, 1 μg of 4-1BB-ODN conjugate was incubated with increasing molar excess of cODN-AlexaFluor-647. Following hybridization, the reaction mixture was incubated with 10 μL of 4-1BB DynaBeads for 10 minutes at room temperature. The beads were washed in 1 mL of FACS Buffer (PBS + 2% FBS) and analyzed by flow cytometry as described. As a negative control, an unrelated antibody (mPD-1 clone RMP1-14; BioXCell)-ODN conjugate was used to verify the specificity of the 4-1BB-ODN antibody binding to the bead.

### ODN Sequences:

The following ODN sequences were used in the study, as specified in the figure legends. All ODNs were obtained from Integrated DNA Technologies or Trilink BioTechnologies, Inc. Where noted, 2’ O-methylated (m) pyrimidines or phosphorothioate (*) modified oligos were utilized to enhance stability.

Duplexed Tap 2 siRNA (Sense: 5′-GmCmUGmCAmCAmCGGmUmUmCAGAAmU; Antisense: 5′-AUUCUGAACCGUGUGCAGCmUmU) or Scrambled Control (Sense: 5′-mUAAAGAAmCmCAmUGGmCmUAAmCmC; Antisense: 5′ GGUUAGCCAUGGUUCUUUAmUmU) were delivered to target cells via conjugation to CpG. CpG 1668 (5′-T*C*C*A*T*G*A*C*G*T*T*C*C*T*G*A*T*G*C*T*), extended at the 3′ end with a 12-carbon spacer followed by the linker 5′-mCGAGGmCmUAmUmCmUAGAAmUGmUAmC, was annealed to a complementary sequence on the Tap 2 siRNA as described above. This construct was used to deliver Tap 2 siRNA to TLR9-expressing cells. Annealing was performed at an equimolar ratio of the complementary sequences in PBS with Ca^2+^ and Mg^2+^. The mixture was heated for 10 minutes at 85 °C in a heat block, then allowed to cool to room temperature before being stored at −80 °C until use.

Antibodies were used to deliver KLF2 siRNA, control scrambled siRNA ODNs by annealing to the ODN (5′-/5AmMC6/rAmUrAmGmUrAmCrAmUmUmCmUrArGrAmUrArGmCmC) labeled antibody with a 3′ complementary linker separated from the sequence by a nine-carbon spacer and was used to deliver siRNA to 4-1BB-expressing T cells. Annealing was carried out at equimolar ratio of the complementary sequences in PBS with Ca^2+^ Mg^2+^ for 10 min at 85 °C in a heat block and allowed to cool to room temperature and stored at − 80 °C until use.

KLF2 siRNA (Sense: 5’-rGmCrGrGmCrArArGrAmCmCmUrAmCrAmCmCAA; Antisense: 5’-rUrUrGrGrUrGrUrArGrGrUrCrUrUrGrCrCrGrCmUmU).

### Flow Cytometry Analysis:

Tumor-infiltrating lymphocytes (TILs) or tumor cells were analyzed as previously described. Mice were euthanized, and spleens and tumors were removed at timepoints indicated in figure legends. Tumors were dissected into small pieces and incubated with enzyme mix of Mouse tumor dissociation kit (Miltenyi Biotec) in RPMI-1640 medium and then dissociated using the gentleMACS Dissociator (Miltenyi Biotec) according to the manufacturer’s instructions. For spleen, single cell suspension was prepared by meshing the spleen on 70 μm nylon strainer by using the flat plunger end of a syringe. The resulting cell suspension was filtered through a 70 μm nylon strainer to obtain a single-cell population. Red blood cells were lysed using ACK Buffer (ThermoFisher), and cells were washed twice with FACS buffer (PBS pH 7.2, supplemented with 0.5% BSA, 2 mM EDTA, and 0.09% azide) and stained according to the described protocol. Cells were incubated with Fc-blocking antibodies (purified anti-mouse CD16/32, clone 93, Biolegend) for 10 min at 4 °C, then incubated with antibodies cocktail diluted in Brilliant Stain Buffer (BD Biosciences) for 30 min at 4 °C. Subsequently, Cell suspensions were incubated with fixable viability dye Live-Dead Blue (ThermoFisher Scientific) for 15 min at RT. After washing cells with PBS twice, cells were fixed with BD Cytofix/Cytoperm according to the manufacturer’s instructions. After cell permeabilization, intracellular staining was performed by incubating intracellular antibody for 30 mins at RT. Cells were analyzed using a Beckman Coulter’s CytoFLEX flow cytometer or BD’s (Becton Dickinson) LSRFortessa cytometer (Flow Cytometry Shared Resource; UMiami Sylvester Cancer Center) and data were analyzed using FCS express (De Novo software).

Multicolor flow cytometry staining was performed using the following antibodies: CD45-FITC (I3/2.3), CD69-AF647 (H1.2F3), CD49a-PECy7 (HMα1), CD103-BV421 (2E7), CD49b-PEDazzle594 (DX5), CD3-BV711 (17A2), CD4-AF700 (RM4-5), CD8a-BV510 (53-6.7), CD44-PerCP-Cy5.5 (IM7), CD62L-BV605 (MEL-14) CCR7-BV650 (4B12), CD127-BV711 (SB/199), KLRG1-BV605 (2F1/KLRG1) all obtained from BioLegend. CD3-BUV737 (17A2), CD4-BUV395 (RM4-5), CD8a-BB700 (53-6.7), CD44-BV786 (IM7), CD62L-APC Cy7 (MEL-14), CD19-BUV805 (1D3) all purchased from BD Biosciences. KLF2-PE purchased from LS Bio.

### Mice:

All animal work was conducted with the approval of the University of Miami Institutional Animal Care and Use Committee (IACUC) and adhered to federal, state, and local guidelines. Female C57Bl/6 mice, aged 10–12 weeks, were used for all studies and were purchased from The Jackson Laboratory.

### Tumor Models:

In the RMA-S T lymphoma model, 7 to 9-week-old female C57BL/6 mice were injected subcutaneously in the right flank with 4 × 10^5^ RMA-S tumor cells on day 0. Treatments were administered on days 3 and 6, with a single dose of 0.75 nmol CpG-siRNA conjugate (1.3 mg/kg) injected subcutaneously near the inguinal lymph node in the right flank. On day 4, 5, 7 and 8, mice were treated with four doses of 100 μg of antibody-siRNA/ODN injected intraperitoneally. Tumor volumes were measured every other day using digital calipers. Two orthogonal dimensions were recorded with larger dimension as tumor length, and the dimension at a 90° angle as tumor width. Tumor volume was calculated using the formula: Tumor volume = 0.5 × Length × Width^2^. Tumor volume reaching 1000 mm^3^ or tumor ulceration was used as the experimental endpoint.

In the B16 tumor model, 7 to 9-week-old female C57BL/6 mice were injected subcutaneously in the right flank with 5 × 10^4^ RMA-S tumor cells on day 0. GM-CSF-expressing B16/F10.9 tumor cells, provided by G. Dranoff, were irradiated (35 Gy) and 2 × 10^6^ cells were injected subcutaneously at days 3 and 6. On days 4, 5, 7, and 8, mice were treated with four doses of 100 μg of antibody-siRNA/ODN administered via intraperitoneal injection. Tumor volumes were measured every other day using digital calipers as described earlier. Tumors reaching a volume of 1000 mm^3^ or showing ulceration were considered the experimental endpoint.

#### Depletion of CD4+ and CD8+ cells.

CD4-specific antibody (catalog #BE0003-1, clone GK1.5) and CD8a-specific antibody (catalog #BE0117, clone 53-6.7) from Bio X Cell were used to deplete CD4^+^ and CD8^+^ T cells, respectively. In the B16 tumor model, C57BL/6J mice received intraperitoneal injections of anti-CD4 or anti-CD8a antibodies (100 μg/100 μL) starting on day 3 after tumor implantation, followed by injections every 7 days. GM-CSF-expressing B16/F10.9 tumor cells were irradiated with 35 Gy and 2 × 10^6^ and injected subcutaneously on days 3 and 6. Mice were treated with four doses of 100 μg of antibody-siRNA/ODN via intraperitoneal injection on days 4, 5, 7, and 8.

### CD8 T cell Isolation and qPCR Analysis

Splenocytes were isolated from the spleens of healthy 6–8-week-old C57BL6/J mice. Under sterile conditions, the spleens were excised and crushed using a syringe plunger through a 70μm cell strainer. Red blood cells were lysed with ACK Buffer (ThermoFisher), followed by two washes with sterile PBS containing 2% FBS and 1 mM EDTA. CD8^+^ T cells were then isolated from the splenocytes using the EasySep^™^ Mouse CD8+ T Cell Isolation Kit (Stemcell Technologies) according to the manufacturer’s protocol. Isolated CD8^+^ T Cells were subsequently plated in 24 well plate at a density of 1 × 10^6^ cells per well per ml of cel culture medium (RPMI Medium 1640 with 2 mM L-Glutamine, 10% FBS, 100 U/mL penicillin/streptomycin, 1000U/ml rIL-2). Subsequently, CD8+ T cells were polyclonally activated with CD23/CD28 antibodies and incubated with 4-1BB antibodies conjugated with either KLF2-siRNA or control siRNA. After 72hr, cells were harvested, and RNA was extracted using RNeasy kit (Qiagen) as per manufacturer’s protocol. Expression of KLF-2, S1PR1 and CD62L was determined by qRT-PCR. RNA was quantified using an Thermo Scientific nanodrop 1000 UV/VIS Spectrophotometer. cDNA synthesis was carried out with the High-Capacity cDNA Reverse Transcription Kit (Applied Biosystems). For each TaqMan qPCR assay, 25–50 ng cDNA equivalents of mRNA were used per reaction, using a Step One qPCR machine (Applied Biosystems). TaqMan probes, ordered from Thermo Fisher Scientific, corresponding to the genes of interest or housekeeping genes, included: KLF2 (Mm00500486_g1), ACTB (Mm02619580_g1), S1pr1 (Mm02619656_s1), and CD62L (Mm00441291_m1)

### Statistical analysis:

Data analysis was performed using Prism 7.0 (GraphPad). All experiments were repeated as described in the figure legends. Statistical significance among multiple groups was determined using one-way ANOVA followed by Dunnett’s multiple comparison test, while comparisons between two groups were performed using an unpaired Student’s t-test. Tumor survival data were analyzed using the Kaplan-Meier method, and survival curves were compared using the Mantel-Cox log-rank test. Error bars represent the standard error of the mean (SEM), and a p-value of less than 0.05 was considered statistically significant.

## Data Availability

All data generated in this study are included in this published article. For additional inquiry contact Eli Gilboa at egilboa@med.miami.edu

## References

[1] Park S. L., Gebhardt T. & Mackay L. K. Tissue-Resident Memory T Cells in Cancer Immunosurveillance. Trends Immunol 40, 735–747 (2019). 10.1016/j.it.2019.06.002 31255505

[2] Amsen D., van Gisbergen K., Hombrink P. & van Lier R. A. W. Tissue-resident memory T cells at the center of immunity to solid tumors. Nat Immunol 19, 538–546 (2018). 10.1038/s41590-018-0114-2 29777219

[3] Szabo P. A., Miron M. & Farber D. L. Location, location, location: Tissue resident memory T cells in mice and humans. Sci Immunol 4 (2019). 10.1126/sciimmunol.aas9673 PMC6778482 30952804

[4] Masopust D. & Soerens A. G. Tissue-Resident T Cells and Other Resident Leukocytes. Annu Rev Immunol 37, 521–546 (2019). 10.1146/annurev-immunol-042617-053214 30726153 PMC7175802

[5] Steinert E. M. Quantifying Memory CD8 T Cells Reveals Regionalization of Immunosurveillance. Cell 161, 737–749 (2015). 10.1016/j.cell.2015.03.031 25957682 PMC4426972

[6] Gebhardt T. Memory T cells in nonlymphoid tissue that provide enhanced local immunity during infection with herpes simplex virus. Nat Immunol 10, 524–530 (2009). 10.1038/ni.1718 19305395

[7] Jiang X. Skin infection generates non-migratory memory CD8+ T(RM) cells providing global skin immunity. Nature 483, 227–231 (2012). 10.1038/nature10851 22388819 PMC3437663

[8] Shin H. & Iwasaki A. A vaccine strategy that protects against genital herpes by establishing local memory T cells. Nature 491, 463–467 (2012). 10.1038/nature11522 23075848 PMC3499630

[9] Rotrosen E. & Kupper T. S. Assessing the generation of tissue resident memory T cells by vaccines. Nat Rev Immunol 23, 655–665 (2023). 10.1038/s41577-023-00853-1 37002288 PMC10064963

[10] Gavil N. V., Cheng K. & Masopust D. Resident memory T cells and cancer. Immunity 57, 1739–1751 (2024). 10.1016/j.immuni.2024.06.017 PMC11529779 39142275

[11] Skon C. N. Transcriptional downregulation of S1pr1 is required for the establishment of resident memory CD8+ T cells. Nat Immunol 14, 1285–1293 (2013). 10.1038/ni.2745 24162775 PMC3844557

[12] Gebhardt T., Palendira U., Tscharke D. C. & Bedoui S. Tissue-resident memory T cells in tissue homeostasis, persistent infection, and cancer surveillance. Immunol Rev 283, 54–76 (2018). 10.1111/imr.12650 29664571

[13] Kaech S. M., Wherry E. J. & Ahmed R. Effector and memory T-cell differentiation: implications for vaccine development. Nat Rev Immunol 2, 251–262 (2002). 10.1038/nri778 12001996

[14] Hinrichs C. S., Gattinoni L. & Restifo N. P. Programming CD8+ T cells for effective immunotherapy. Current opinion in immunology 18, 363–370 (2006). 10.1016/j.coi.2006.03.009 16616471 PMC1540013

[15] Klebanoff C. A., Gattinoni L. & Restifo N. P. CD8+ T-cell memory in tumor immunology and immunotherapy. Immunological reviews 211, 214–224 (2006). 10.1111/j.0105-2896.2006.00391.x 16824130 PMC1501075

[16] Iborra S. Optimal Generation of Tissue-Resident but Not Circulating Memory T Cells during Viral Infection Requires Crosspriming by DNGR-1(+) Dendritic Cells. Immunity 45, 847–860 (2016). 10.1016/j.immuni.2016.08.019 27692611 PMC5074364

[17] Ramirez D. E., Mohamed A., Huang Y. H. & Turk M. J. In the right place at the right time: tissue-resident memory T cells in immunity to cancer. Curr Opin Immunol 83, 102338 (2023). 10.1016/j.coi.2023.102338 37229984 PMC10631801

[18] Liikanen I. Hypoxia-inducible factor activity promotes antitumor effector function and tissue residency by CD8+ T cells. J Clin Invest 131 (2021). 10.1172/JCI143729 PMC8011896 33792560

[19] Milner J. J. Runx3 programs CD8(+) T cell residency in non-lymphoid tissues and tumours. Nature 552, 253–257 (2017). 10.1038/nature24993 29211713 PMC5747964

[20] Reina-Campos M. Metabolic programs of T cell tissue residency empower tumour immunity. Nature 621, 179–187 (2023). 10.1038/s41586-023-06483-w 37648857 PMC11238873

[21] Enamorado M. Enhanced anti-tumour immunity requires the interplay between resident and circulating memory CD8(+) T cells. Nat Commun 8, 16073 (2017). 10.1038/ncomms16073 28714465 PMC5520051

[22] Cuburu N. Intravaginal immunization with HPV vectors induces tissue-resident CD8+ T cell responses. J Clin Invest 122, 4606–4620 (2012). 10.1172/JCI63287 23143305 PMC3533540

[23] Stary G. VACCINES. A mucosal vaccine against Chlamydia trachomatis generates two waves of protective memory T cells. Science 348, aaa8205 (2015). 10.1126/science.aaa8205 26089520 PMC4605428

[24] Nizard M. Induction of resident memory T cells enhances the efficacy of cancer vaccine. Nat Commun 8, 15221 (2017). 10.1038/ncomms15221 28537262 PMC5458068

[25] Sandoval F. Mucosal imprinting of vaccine-induced CD8(+) T cells is crucial to inhibit the growth of mucosal tumors. Sci Transl Med 5, 172ra120 (2013). 10.1126/scitranslmed.3004888 PMC4086646 23408053

[26] Liu L. Epidermal injury and infection during poxvirus immunization is crucial for the generation of highly protective T cell-mediated immunity. Nat Med 16, 224–227 (2010). 10.1038/nm.2078 20081864 PMC3070948

[27] Wakim L. M., Smith J., Caminschi I., Lahoud M. H. & Villadangos J. A. Antibody-targeted vaccination to lung dendritic cells generates tissue-resident memory CD8 T cells that are highly protective against influenza virus infection. Mucosal Immunol 8, 1060–1071 (2015). 10.1038/mi.2014.133 25586557

[28] Clark E. S., Benaduce A. P., Khan W. N., Martinez O. & Gilboa E. Vaccination against neoantigens induced in cross-priming cDC1 in vivo. Cancer Immunol Immunother 73, 9 (2024). 10.1007/s00262-023-03597-y 38231450 PMC10794404

[29] Kwon B., Lee H. W. & Kwon B. S. New insights into the role of 4-1BB in immune responses: beyond CD8+ T cells. Trends Immunol 23, 378–380 (2002). 12133793 10.1016/s1471-4906(02)02263-9

[30] Wang C., Lin G. H., McPherson A. J. & Watts T. H. Immune regulation by 4-1BB and 4-1BBL: complexities and challenges. Immunol Rev 229, 192–215 (2009). 19426223 10.1111/j.1600-065X.2009.00765.x

[31] Mueller S. N. & Mackay L. K. Tissue-resident memory T cells: local specialists in immune defence. Nat Rev Immunol 16, 79–89 (2016). 10.1038/nri.2015.3 26688350

[32] Garrido G. Vaccination against Nonmutated Neoantigens Induced in Recurrent and Future Tumors. Cancer Immunol Res 8, 856–868 (2020). 10.1158/2326-6066.CIR-20-0020 32295785 PMC7339786

[33] Baeyens A., Fang V., Chen C. & Schwab S. R. Exit Strategies: S1P Signaling and T Cell Migration. Trends Immunol 36, 778–787 (2015). 10.1016/j.it.2015.10.005 26596799 PMC4832571

[34] Virassamy B. Intratumoral CD8(+) T cells with a tissue-resident memory phenotype mediate local immunity and immune checkpoint responses in breast cancer. Cancer Cell 41, 585–601 e588 (2023). 10.1016/j.ccell.2023.01.004 36827978

